# Enhanced diffusion through multivalency†

**DOI:** 10.1039/d4sm00778f

**Published:** 2024-12-04

**Authors:** Ladislav Bartoš, Mikael Lund, Robert Vácha

**Affiliations:** a CEITEC – Central European Institute of Technology, Kamenice 753/5 625 00 Brno Czech Republic; b National Centre for Biomolecular Research, Faculty of Science, Masaryk University, Kamenice 753/5 625 00 Brno Czech Republic robert.vacha@muni.cz; c Division of Computational Chemistry, Lund University Sweden mikael.lund@teokem.lu.se; d LINXS Institute of Advanced Neutron and X-ray Science, Lund University Sweden; e Department of Condensed Matter Physics, Faculty of Science, Masaryk University, Kotlářská 267/2 611 37 Brno Czech Republic

## Abstract

The diffusion of macromolecules, nanoparticles, viruses, and bacteria is essential for targeting hosts or cellular destinations. While these entities can bind to receptors and ligands on host surfaces, the impact of multiple binding sites—referred to as multivalency—on diffusion along strands or surfaces is poorly understood. Through numerical simulations, we have discovered a significant acceleration in diffusion for particles with increasing valency, while maintaining the same overall affinity to the host surface. This acceleration arises from the redistribution of the binding affinity of the particle across multiple binding ligands. As a result, particles that are immobilized when monovalent can achieve near-unrestricted diffusion upon becoming multivalent. Additionally, we demonstrate that the diffusion of multivalent particles with a rigid ligand distribution can be modulated by patterned host receptors. These findings provide insights into the complex diffusion mechanisms of multivalent particles and biological entities, and offer new strategies for designing advanced nanoparticle systems with tailored diffusion properties, thereby enhancing their effectiveness in applications such as drug delivery and diagnostics.

## Abstract 2

Increasing the number of binding sites on particles, while keeping the same surface affinity, enhances particle diffusion along surfaces.

## Graphical abstract

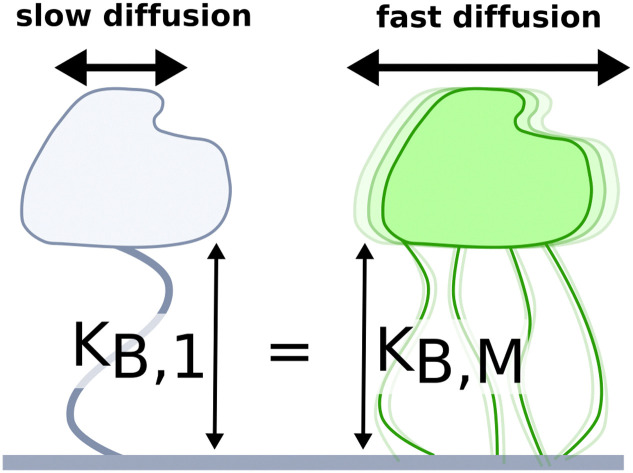

## Introduction

Multivalent binding refers to the simultaneous non-covalent interaction of one entity with multiple binding ligands on another entity. This mode of interaction is frequent in biological systems, involving diverse entities such as proteins,^1–3^ antibodies,^4^ carbohydrates,^5–7^ and viruses,^8–10^ but also in colloids, polymers, and nanoparticles.^11–13^

The unique characteristic of multivalent binding lies in its ability to amplify the strength and specificity of molecular recognition. By engaging multiple receptor sites, a molecule can enhance its binding affinity and selectivity towards its target,^14,15^ enabling biological processes such as cell adhesion,^16,17^ signaling,^18,19^ immune response modulation,^20,21^ or viral entry.^8^

For instance, *Escherichia coli* binds to epithelial cells through multivalent interactions between the FimH adhesin on its pili and the mannose groups on oligosaccharides on the cell surface.^22–24^ The entry of herpesviruses into cells is facilitated by multivalent interactions between multiple viral glycoproteins and cell receptors,^25^ and is largely dependent on the ability of the virion to diffuse on the cell surface.^9^ Multivalency has also been suggested to be necessary for the movement of influenza virus through extracellular mucus.^26^

In this study, we present a simple numerical model of particles diffusing/walking on host surfaces or tethers to provide the relationship between the valency of the particles and their diffusion rates. We restrict the study to particles with roughly equal affinity towards the host, and demonstrate that increased valency dramatically accelerates diffusion. In addition, we show the impact of the rigid distribution/geometry of host receptors on the diffusion rates of the particles with matching distribution of ligands.

## Methods

**Fig. 1:**
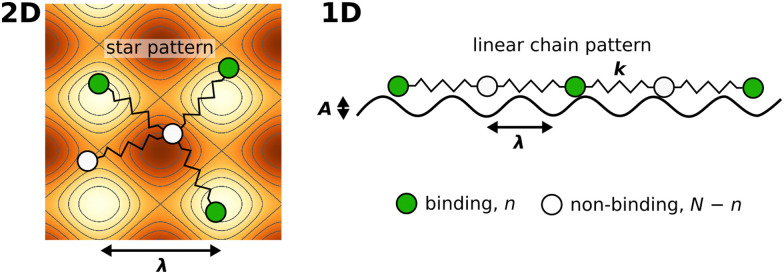
Illustration of the employed model. The diffusing particle was composed of *N* ligands connected *via* linkers of specific stiffness *k*. *n* of the ligands were binding, *i.e.* interacting with the surface potential, which corresponds to the valency of the particle. In the 2D case, the surface potential represented a host surface such as a cell membrane with uniformly distributed receptors. In the 1D case, the potential represented a tether such as a microtubule or a DNA double helix. The distance between the receptors was specified by the parameter *λ* while the affinity of the particle to the surface was set by the parameter *K*_B_ = *An*.

The numerical model consisted of *N* diffusing point-like ligands connected by linkers represented by harmonic springs with force constant *k* and equilibrium distance *λ*_eq_, the later defining the unit length. In the following text, we refer to a group of linked ligands as a particle. All particles were able to translate either on a line (1D) or on a plane (2D) decorated with an oscillating potential given by1
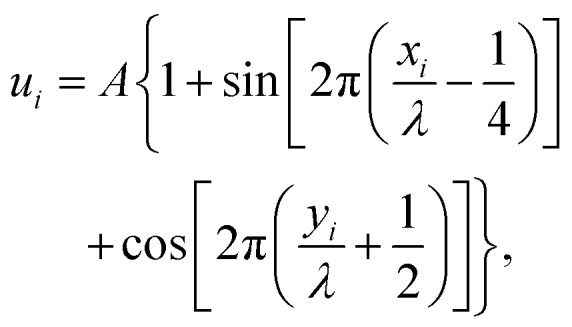
where {*x*_*i*_, *y*_*i*_} is the *i*’th ligand position (for 1D, *y*_*i*_ = 0), *A* is the amplitude, *λ* is the distance between the potential wells (see Fig. 1). See eqn (S1) (ESI†) for the full Hamiltonian of the simulated systems.

The potential wells represented the receptor sites on the host surface and the amplitude (*A*) determined the affinity of the ligand to the receptors. Only *n* ≤ *N* of the *N* ligands interacted with the potential whereby the cumulative ligand affinity was set *K*_B_ = *An* for varying *n*. The *A* was scaled with *n* to keep *K*_B_ constant, ensuring that the particle's overall affinity to the surface remained roughly the same regardless of its valency. Two connectivity patterns between the ligands were simulated: a linear chain and a star, where all ligands were connected to one central ligand (see Fig. 1). See Table 1 and Table S2 (ESI†) for detailed information about the simulated patterns.

**Table 1:** Standard, quasi-homogeneous distribution of ligands used for the presented simulations, unless stated otherwise. 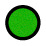 corresponds to a ligand that interacts with the surface potential (binds to receptors), while ○ corresponds to a non-binding ligand

	*N* = 5		*N* = 10
	1D	2D	1D
*n* = 0	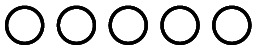	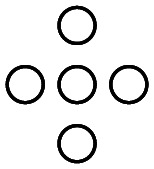	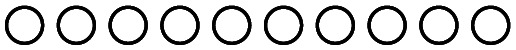
*n* = 1	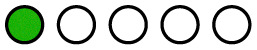	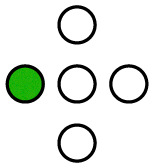	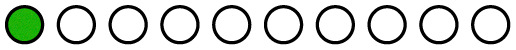
*n* = 2	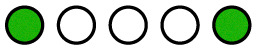	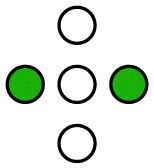	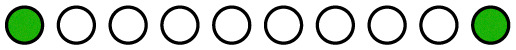
*n* = 3	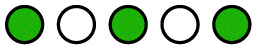	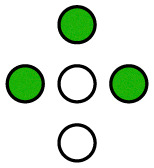	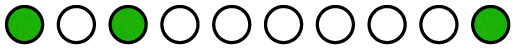
*n* = 4	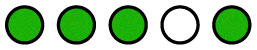	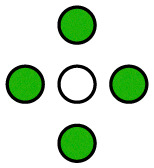	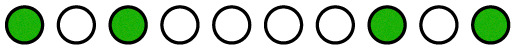
*n* = 5	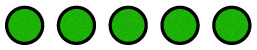	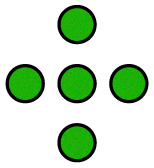	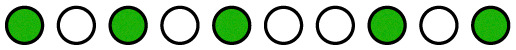
*n* = 6	N/A	N/A	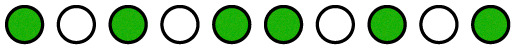
*n* = 7	N/A	N/A	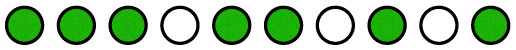
*n* = 8	N/A	N/A	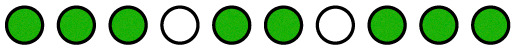
*n* = 9	N/A	N/A	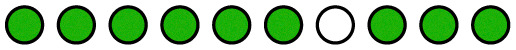
*n* = 10	N/A	N/A	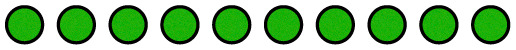

The system was propagated using dynamic Monte Carlo (MC) where individual ligands were moved using MC translation moves, accepted or rejected according to Metropolis–Hastings criterion.^27^ The maximum displacement was set to 0.1*λ*_eq_ which also defined the time-scale of the simulation. The maximum displacement was sufficiently small, preventing the ligands from jumping between potential energy wells without crossing the barriers. All energies are reported in kT. The code used to perform the simulations is available from doi.org/10.5281/zenodo.8340209.

Initially, every replica of each system was equilibrated for either 50 000 (most systems) or 200 000 (systems with *N* = 10, *n* = 1, and *k* ≤ 10) sweeps, followed by 30 000 production sweeps. For each sweep, *N* translational attempts were performed. Every 100 sweeps, we calculated the mean-square deviation (MSD) between the current center of geometry of the diffusing ligands and their center of geometry at the start of the simulation. 3000 of these independent replicas were used to collect MSD data and construct a single MSD curve. A line was then fitted through the production part of the collected MSD curve and from the slope of the fitted line, we obtained an estimate of the diffusion rate (diffusion coefficient). We repeated the entire process of collecting MSD data 10 times and averaged the calculated diffusion rates. The error was taken as a standard deviation of the calculated diffusion rates. See Fig. S1 (ESI†) for an example of MSD curves calculated for a selected system visualizing how the diffusion coefficients are obtained and Fig. S2 (ESI†) for an example of more MSD curves calculated for diverse systems showing that the diffusion in the production phase is always normal.

In total, each system was simulated with 30 000 independent replicas, resulting in 2.4 (most systems) or 6.9 (*N* = 10, *n* = 1, *k* ≤ 10) billion MC sweeps being performed for each system. The calculated diffusion rates, *D*, were normalized with the corresponding free diffusion, *D*_0_, in the absence of any surface potential. The obtained relative diffusion rate *D*/*D*_0_ thus depicts how much slower the diffusion of the bound particle was compared to the diffusion of free particle.

## Results

**Fig. 2:**
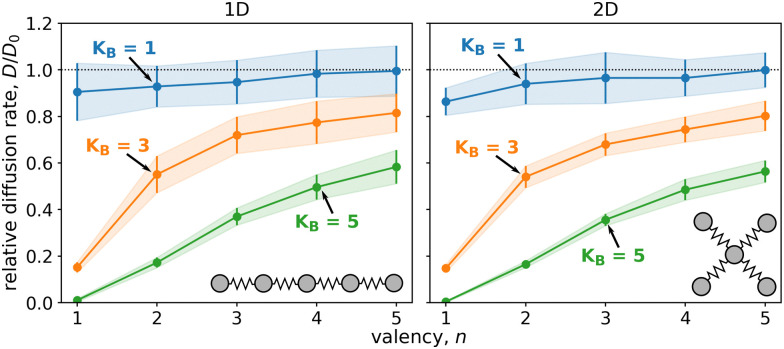
The diffusion of particles comprised of five ligands (*N* = 5) with varying valency, *i.e.* the number of binding ligands, *n*, ranging from 1 to 5, and three different cumulative ligand affinities, *K*_B_. Linkers represented by harmonic bonds with a force constant of *k* = 1 connected the ligands, with the distance between the potential wells of the surface equivalent to the equilibrium linker lengths. The relative diffusion rates, *D*/*D*_0_, represent the diffusion of bound particle with respect to freely diffusing particle without any surface potential. The left plot depicts the diffusion of a linear chain on a line, while the right plot is for star arrangement of ligands diffusing on a plane.

We began by examining the impact of multiple weakly binding ligands compared to a single strongly binding ligand. Fig. 2 displays the diffusion of particles composed of five ligands that form either a linear chain (1D) or a star (2D). Each particle had a different valency, *n*, *i.e.* a varying number of binding ligands. We calculated diffusion rates for particles with three different cumulative ligand affinities, *K*_B_, which remained the same regardless of *n* and represent an estimate of the binding free energy of the particles. The diffusion rates are displayed as relative values to the unbound particles, *i.e.* free diffusion in the absence of surface potential.

As expected, particles with stronger cumulative ligand affinity, *K*_B_, diffused less than those with weaker affinity. (Specifically, particles with *K*_B_ = 5 exhibited lower diffusion rates than particles with *K*_B_ = 1.)

**Fig. 3:**
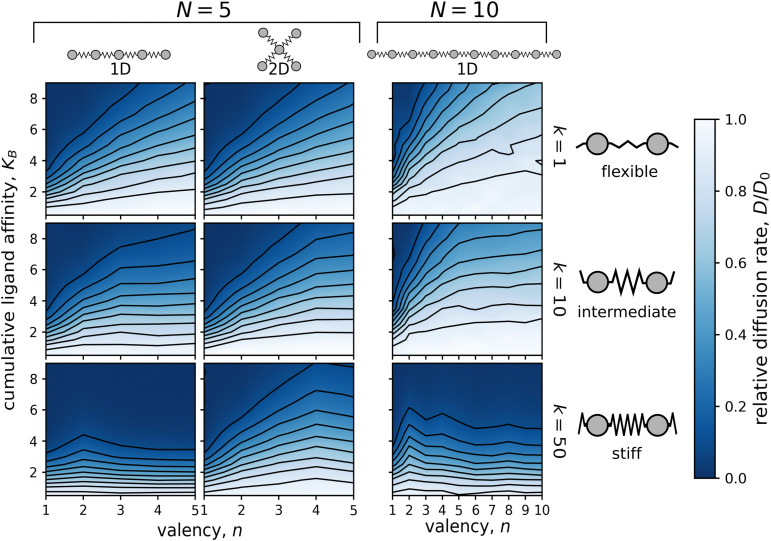
Dependence of the relative diffusion rate, *D*/*D*_0_, on the valency, *n*, and the cumulative ligand affinity, *K*_B_. Simulations were performed on particles consisting of either *N* = 5 (two columns on the left) or *N* = 10 ligands (column on the right) connected by flexible (*k* = 1; top row), intermediate (*k* = 10; middle row), or stiff (*k* = 50; bottom row) linkers. The distance between the surface potential wells matched the equilibrium linker lengths, *i.e. λ*/*λ*_eq_ = 1.0. Diffusion of particles with *N* = 5 was calculated in both 1D (linear chain) and 2D (star-shaped) geometries, while diffusion of particles with *N* = 10 was examined only in the 1D case (linear chain). One simulation was run for each integer *n* and each increment of 0.5 in *K*_B_.

The relative diffusion rate was dramatically affected by valency, *n*. Increasing the number of binding ligands led to faster diffusion for all affinities when compared to the particle with only one binding ligand (*n* = 1). Note that with increasing valency the diffusion rate seems to be converging to free diffusion, *D*_0_. This trend is especially evident for particles with 10 ligands, as shown in Fig. 3 and Fig. S3 (ESI†).

The correlation between the valency, *n*, and the relative diffusion rate, *D*/*D*_0_, applied to all studied cumulative ligand affinities, as demonstrated in Fig. 3. This relationship was particularly pronounced for particles containing ten ligands, where the affinity per ligand, *A* = *K*_B_/*n*, could decrease significantly more than for the 5-ligand particles. For 10-ligand particles with flexible linkers (*k* = 1), it was possible to increase the diffusion rate from nearly immobile (*D*/*D*_0_ < 0.1) to almost as rapid as a non-binding particle (*D*/*D*_0_ ≈ 0.9) by redistributing the affinity from one to ten binding ligands.

It should be noted that while the cumulative ligand affinity of the particle to the surface was maintained, the binding free energy did not necessarily remain constant as the valency increased. As shown in Fig. S6 (ESI†), for particles with *k* = 1 and *k* = 10, the binding free energy—representing the actual affinity of the particle toward the surface—was the most negative (strongest) for monovalent particles and gradually became slightly weaker as the valency, *n*, increased. However, we also prepared several systems where the binding free energy was kept constant (see Table S1, ESI†) and observed no significant differences in the trends described in the preceding and the following paragraphs (see Fig. S7, ESI†). Therefore, unless stated otherwise, we chose to present results where the cumulative ligand affinity, rather than the binding free energy, is kept constant, as these conditions are more easily achievable both computationally and experimentally.

In the 1D case, the impact of valency on diffusion was diminished by increasing the stiffness of the linkers connecting the ligands of the particle. In fact, when the force constant of the linkers, *k*, was increased to 50, the diffusion rate became nearly independent of the valency for both *N* = 5 and *N* = 10 particles. The use of stiff linkers resulted in an almost rigid particle with evenly distributed ligands. The spacing between the ligands matched the distance between the surface potential wells. With this “pattern matching”, all binding ligands had to traverse a potential barrier simultaneously leading to the equal height of a diffusion barrier independent of the valency.

The “pattern matching” effect was significantly weaker in two dimensions, regardless of the ligand configuration in the particle (see Fig. S8, ESI†). This is because in 2D each ligand can individually leave the receptor by moving sideways relative to the bond direction. Such sideways movement causes less stretching or contracting of the linkers compared to movement along the bond direction, making it energetically feasible even for very stiff linkers.

**Fig. 4:**
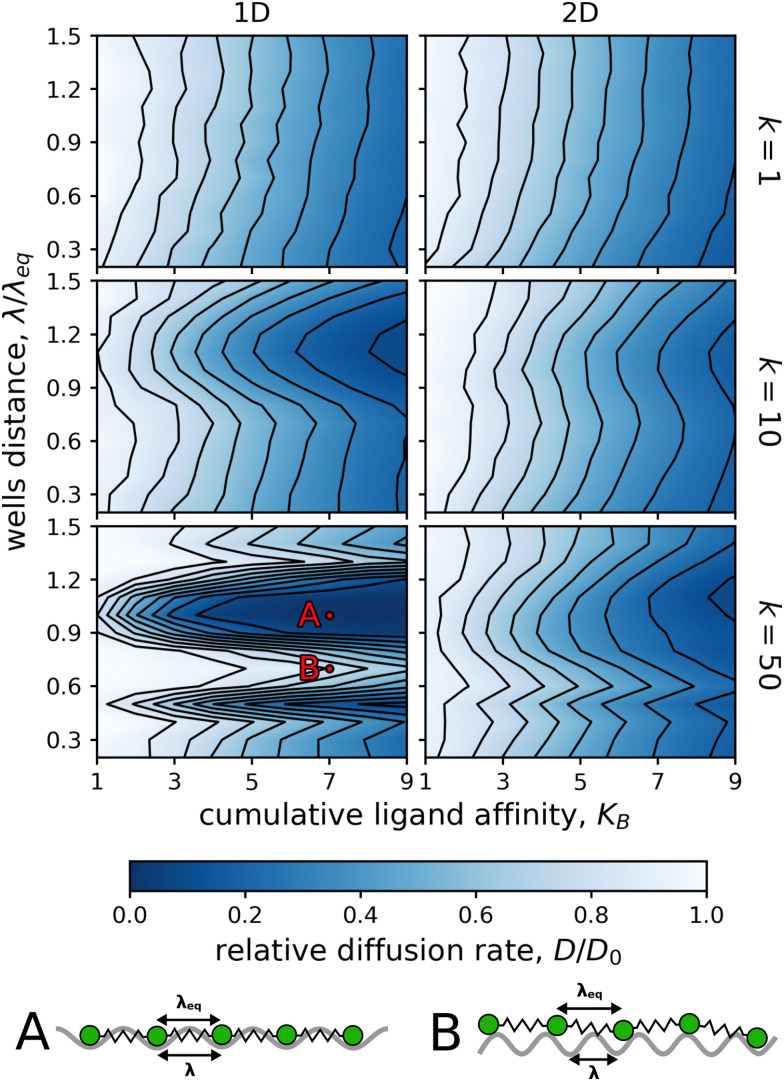
Dependence of the relative diffusion rate, *D*/*D*_0_, on the cumulative ligand affinity, *K*_B_, and the distance between the potential wells, *λ*/*λ*_eq_. The particles were composed of five binding ligands (*N* = 5, *n* = 5), which were connected by flexible (*k* = 1; top row), intermediate (*k* = 10; middle row), or stiff (*k* = 50; bottom row) linkers. The ligands were arranged either as a linear chain (1D) or as a star (2D). One simulation was run for each increment of 1.0 in *K*_B_ and each increment of 0.1 in *λ*/*λ*_eq_. (A) Schematic representation of a linear chain which ligands are all positioned in the potential surface wells as *λ*/*λ*_eq_ = 1.0. In such case, diffusion is not enhanced with the increasing valency. (B) Schematic representation of a linear chain with linkers mismatching the potential surface pattern as *λ*/*λ*_eq_ = 0.7. In such case, the diffusion is enhanced with increasing valency.

To examine the “pattern matching” effect in more detail, we investigated the relationship between the equilibrium linker length and the potential wells distance and their effect on the diffusion rate. We assessed the diffusion rates of particles with *N* = 5 and *n* = 5 in both 1D and 2D space for various distances between the potential wells. As depicted in Fig. 4, particles with flexible linkers (*k* = 1) were mostly unaffected by the altered distance between the potential wells.

In contrast, the diffusion of particles with intermediately flexible linkers (*k* = 10) was significantly reduced when the distance between the wells of the surface potential roughly matched the equilibrium length of the linkers connecting the ligands.

Interestingly, the relative diffusion rate had its minimum at a distance of *λ*/*λ*_eq_ = 1.1, instead of the anticipated 1.0, indicating that the particle diffused the slowest when the distance between the potential wells was slightly longer than the equilibrium linker length. The origin of this effect is entropic and relates to the system setup, further information is in the ESI.†

Particles with stiff linkers (*k* = 50) showed a significant reduction in diffusion not only at wells distances matching the linker lengths but also at *λ*/*λ*_eq_ ≈ 0.5, which was especially noticeable in the 1D case. This behavior was unsurprising since at this well spacing, all binding ligands also need to be simultaneously at the potential barriers for further diffusion.

**Fig. 5:**
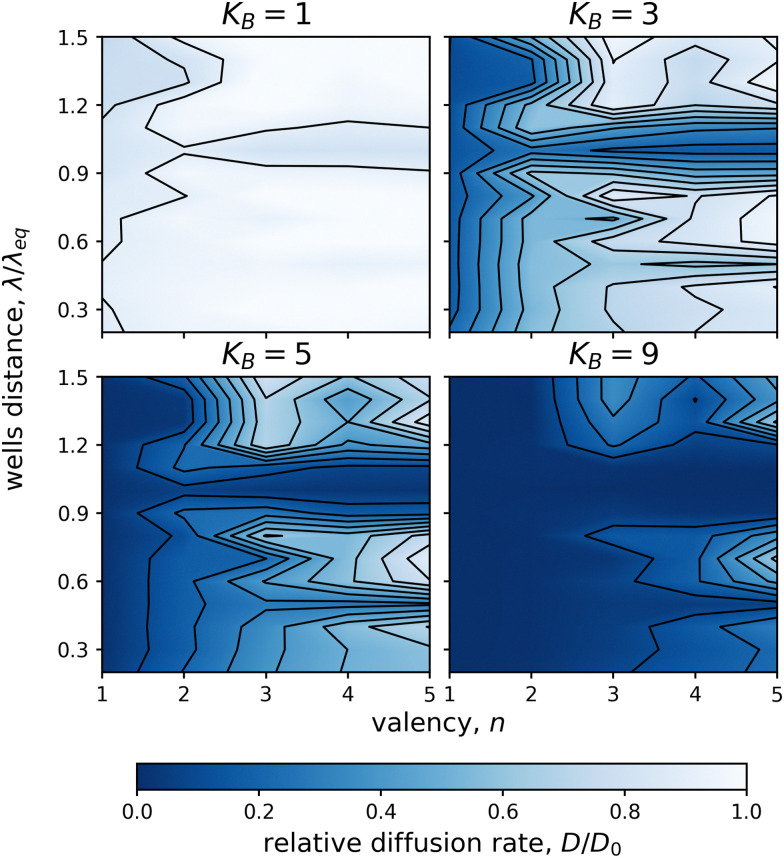
Dependence of the 1D relative diffusion rate, *D*/*D*_0_, on valency, *n*, and potential wells distance, *λ*/*λ*_eq_. Four cumulative ligand affinities, *K*_B_ ∈ {1, 3, 5, 9}, were used. All ligands were connected with stiff linkers (*k* = 50). Note that if the wells distance matched the linker length *λ*/*λ*_eq_ ≈ 1.0, the relative diffusion rate was independent of the valency. Similar, but weaker, effect was observed also for *λ*/*λ*_eq_ ≈ 0.5. One simulation was run for each integer *n* and each increment of 0.1 in *λ*/*λ*_eq_.

Furthermore, in Fig. 5, we show that increasing the valency of particles with stiff linkers (*k* = 50) had almost no impact on the relative diffusion rate if the equilibrium linker length roughly matched the wells distance. The same effect is seen in Fig. 3. Moreover, the effect of “pattern matching” can be modulated by using a surface potential with “nonmatching” wells distances. For instance, *λ*/*λ*_eq_ = 0.7 can lead to increased effect of valency for stiffly linked ligands, as shown in Fig. S4 (ESI†).

**Fig. 6:**
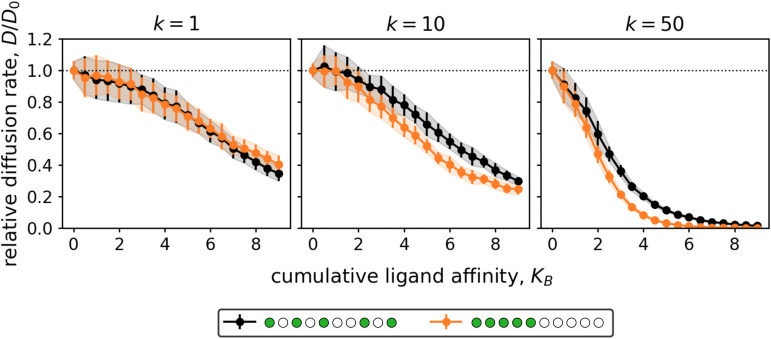
The effect of binding ligand distribution on 1D diffusion. Particles consisting of *N* = 10 ligands, 5 of which were binding (*n* = 5), connected by flexible (*k* = 1), intermediate (*k* = 10), or stiff (*k* = 50) linkers were studied. The distance between the surface potential wells matched the equilibrium linker lengths. The black lines represent the quasi-homogeneous distribution of binding ligands used for most simulations, while the orange lines correspond to the alternative distribution in which all binding ligands were concentrated at one end of the chain. One simulation was run for each increment of 0.5 in *K*_B_. Note that for sufficiently tightly linked particles (*k* ≥ 10), the concentrated pattern of binding ligands led to slightly lower diffusion rates than the quasi-homogeneous pattern. This was not observed for particles with *k* = 1 as these ligands were so weakly linked that they could exchange their positions in the chain.

Additionally, we showed that the observed increase of the relative diffusion rate with increasing valency is independent of the distribution of binding and non-binding ligands within the particles (see Fig. S8 (ESI†)). However, the exact values of the relative diffusion rates may be affected by the distribution of the binding ligands (see Fig. 6).

We also confirmed that the observed behavior is unaffected by the selection of Monte Carlo moves used. Upon incorporating a chain move, which was attempted on average once every sweep, we observed consistent trends with those seen in the absence of the chain move. For results of simulations incorporating a chain move and additional details, see Fig. S9 (ESI†).

Finally, we substituted the sine surface potential given by eqn (1) with an oscillating step surface potential given by eqn (S5) (ESI†), featuring repeating flat valleys with negative potential energy and flat barriers with positive potential energy. For the step surface potential, the previously observed relationship between valency and diffusion persisted unless the width of the barriers became so extensive that all ligands had to traverse it simultaneously. Refer to Fig. S10 (ESI†) for further details.

## Discussion

We conducted dynamic Monte Carlo simulations with a simple model of multivalent particles to investigate their diffusion on a molecular surface and tether. Specifically, we focused on the scenario where multivalent particles bind with varying number of binding ligands to a host surface covered with fixed receptors sites and move laterally without rolling, *i.e.* walking is represented by binding and unbinding of the individual ligands to the receptors. The other mechanisms of diffusion, such as particle rolling, sliding along with attached receptors, and unbinding/rebinding with diffusion in solution have previously been described,^28–32^ but their contribution to diffusion extends beyond the scope of this study.

Our model, designed to simplify the system while being analogous to an ideal gas model, used harmonic bonds to connect ligands, representing the inherent flexibility of the particle including the connection of the ligands. Testing different geometries in 1D and 2D revealed similar behavioral trends, indicating that the connection geometry has minimal impact on the overall behavior.

Our results reveal that particles can have dramatically enhanced surface diffusion with increasing valency. This observation appears to be in contrast with several previous results which established that more bonds between the particle and the host (*i.e.*, increasing valency) lead to a reduction in the diffusion rate.^33–36^ However, in these cases the strength of ligand–receptor bonds remained the same and increasing valency resulted in overall much stronger particle–host adsorption. In our model, we focused on the particles with roughly the same binding affinity to the host (tether or surface). In particular, we decreased the strength of individual ligand–receptor bonds with increasing valency (keeping the valency times ligand–receptor interaction strength constant). Weaker ligand–receptor interactions result in easier unbinding of individual ligands, enabling the particle to move more faster.

Interestingly, Kowalewski *et al.*^33^ presented a general analytical model of multivalent diffusion, assuming unchanged ligand–receptor affinity. Application of this model to systems with maintained overall particle–host affinity predicted slowed diffusion, which starkly contrasted with our simulation results. The discrepancy likely arises because the analytical model was derived without incorporating data from simulations where overall particle affinity remained constant, highlighting the unintuitive behavior of these systems.

Our findings are in line with the reported faster diffusion rate of particles with weaker individual bonds between the ligand and receptors.^37,38^ Moreover, prior research has shown that continuous motion of motor proteins along microtubules relies on multivalency and the individual binding and unbinding of domains.^39^

Additionally, our model predicts that the diffusion rate is significantly reduced for particles with specific pattern/distribution of ligands. This reduction occurs when the ligand pattern matches the pattern of the receptors and the patterns are stiff. In such cases, all binding ligands align with the receptors, resulting in diminished diffusion. This “pattern matching” effect is more pronounced for stiffer patterns and higher valencies, while flexible patterns remain unaffected. Based on these results we anticipate scenarios where the host bound particles exhibit rapid diffusion on surface with heterogeneously distributed receptors until these particles find a matching receptor pattern where the particles are likely to halt.

While we aimed to maintain the overall binding affinity of the particle to the surface regardless of its valency, the actual binding free energy of the particle could slightly vary between particles with different valencies. However, these differences were small, and we have verified that the observed trends remain consistent also when the binding free energy is maintained at a constant level.

In summary, our findings lay the groundwork for designing particles with controlled diffusion capabilities on 1D or 2D targets, including DNA-binding sliding proteins.^40–42^ These insights are be relevant to biological processes that involve multivalent interactions between a host surface and a mobile entity, such as the interactions between cells and viruses or bacteria. Our model may be applicable to various biological systems, including proteins with multiple binding domains separated by unstructured regions or sequences containing sticky residues interspersed with less interactive ones, as well as vesicles, viruses, bacteria, and other cells that can diffuse along protein fibers or the surfaces of organelles, cells, and tissues.

## Conclusions

We demonstrated that multivalent particles can achieve remarkable diffusion acceleration with increasing valency, while bound to a host tether or a surface. By distributing the binding affinity to multiple ligands, the initially non-diffusing monovalent particle could achieve a diffusion rate nearly as high as that of an unbound particle. The fast diffusion could be controlled for particles which have rigid distribution of ligands that matches the distribution of host receptors. Our findings could find applications in various fields utilizing the design of fast-diffusing particles that maintain a strong affinity for target, such as DNA-binding sliding proteins. Additionally, our results could be relevant to biological processes involving the multivalent binding of viruses and bacteria to cells.

## Author contributions

L. B., M. L., and R. V. carried out simulations and analyzed the data. M. L. and R. V. designed the research. L. B., M. L., and R. V. wrote the article.

## Data availability

All simulation data are provided at https://doi.org/10.5281/zenodo.8396688. The code used to perform the simulations is available from https://doi.org/10.5281/zenodo.8340209 (most results), https://doi.org/10.5281/zenodo.13771494 (results presented in Fig. S6 and S7, ESI†), https://doi.org/10.5281/zenodo.10877722 (results presented in Fig. S9, ESI†) and https://doi.org/10.5281/zenodo.10054283 (results presented in Fig. S10, ESI†).

## Conflicts of interest

The authors declare no competing interests.

## Supplementary Material

SM-021-D4SM00778F-s001
