# Supplementary material for: Enhanced diffusion through multivalency
Source: Soft Matter. 2024 Dec 4;21(2):179–85. doi: 10.1039/d4sm00778f (PMC11615653; doi:10.1039/d4sm00778f)
Supplement: SM-021-D4SM00778F-s001 [file SM-021-D4SM00778F-s001.pdf]

Supplementary Information (SI) for Soft Matter.
This journal is © The Royal Society of Chemistry 2024

Supporting Information

Enhanced Diffusion through Multivalency

Ladislav Bartoš,[†,‡] Mikael Lund,[*,¶] and Robert Vácha[*,†,‡,§]

[†] CEITEC – Central European Institute of Technology, Masaryk University, Kamenice 753/5, 625 00 Brno, Czech Republic
[‡] National Centre for Biomolecular Research, Faculty of Science, Masaryk University, Kamenice 753/5, 625 00 Brno, Czech Republic
[¶] Division of Computational Chemistry, Lund University, Sweden
[§] Department of Condensed Matter Physics, Faculty of Science, Masaryk University, Kotlářská 267/2, 611 37 Brno, Czech Republic
* E-mail: mikael.lund@teokem.lu.se, robert.vacha@muni.cz

Hamiltonian of the systems

The full Hamiltonian, H, of the simulated systems is defined as follows:

$$H = \sum_{i=1}^{n} \left[A \left(1 + \sin\left(2\pi \left(\frac{x_i}{\lambda} - \frac{1}{4}\right)\right) + \cos\left(2\pi \left(\frac{y_i}{\lambda} + \frac{1}{2}\right)\right)\right)\right] + \sum_{j=1}^{L} \left[\frac{1}{2} k_j (r_j - \lambda_{\mathrm{eq},j})^2\right] \quad \text{(S1)}$$

where n is the number of binding ligands, A is the amplitude of the oscillating surface potential, λ is the distance between the surface potential wells, $\{x_i, y_i\}$ is the i'th ligand position, L is the number of linkers of the simulated particle, k_j is the force constant of the linker, r_j is the length of the linker, and $\lambda_{\mathrm{eq},j}$ is the equilibrium length of the linker.

Examples of mean-square deviation curves

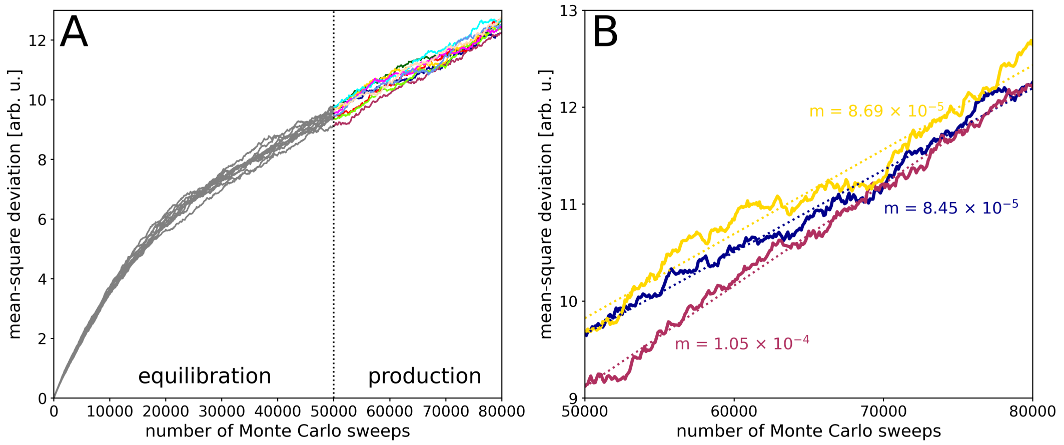

Figure S1: A. Ten mean-square deviation (MSD) curves collected over 30,000 independent simulations of a particle with $N = 5$, $n = 1$, $k = 1$, $\lambda/\lambda_{\rm eq} = 1.0$, $K_B = 3.0$ diffusing on a 1D line. MSD data were collected over the entire simulation run, but data from the first 50,000 sweeps of each replica (equilibration stage) were omitted for line fitting. B. Three selected MSD curves obtained from the same system, displaying fitted lines and their slopes during the production phase of the simulation. The diffusion rate for each curve was calculated by dividing the slope by a scaling factor based on the dimensionality of the problem (2 for 1D or 4 for 2D) and multiplying by an arbitrary constant (1000). The diffusion rates from individual MSD curves were averaged, and their standard deviation represents the error estimate. Throughout the main text, diffusion rates are reported relative to free diffusion calculated using the same method. The line fitting utilized the Rust linreg crate v0.2.0 (crates.io/crates/linreg), while the mean and standard deviation calculations were conducted using the statistical crate v1.0.0 (crates.io/crates/statistical).

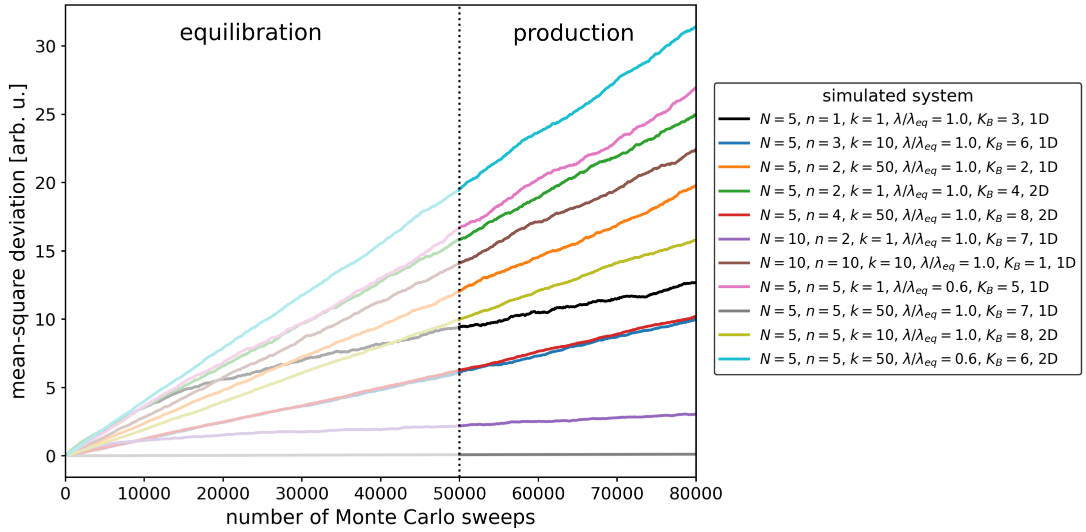

Figure S2: Several randomly selected mean-square deviation (MSD) curves for various diverse systems. Each MSD curve is derived from data collected across 3000 independent simulations. Although some systems exhibit subdiffusive behavior during the equilibration phase, diffusion is always normal during the production phase. The reported diffusion coefficients are calculated solely from the production segment of the MSD curves.

Simplified look at diffusion in systems with particles composed of 10 ligands

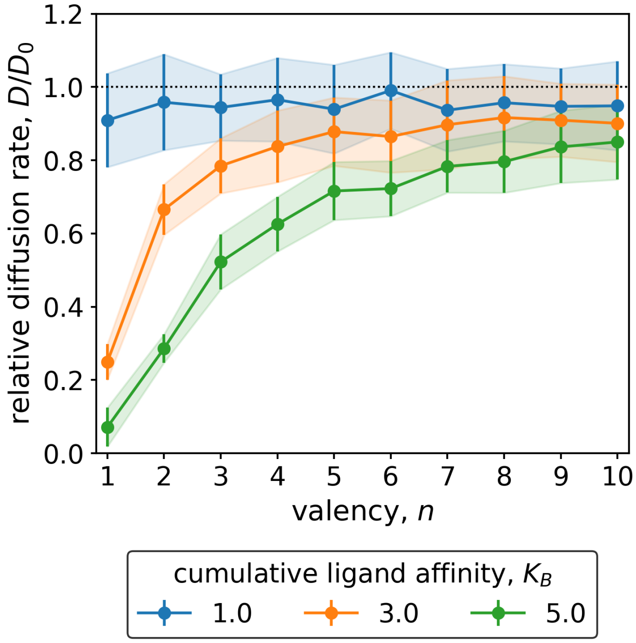

Figure S3: The diffusion of particles composed of ten ligands ($N = 10$) with varying valency, n, ranging from 1 to 10, and three different cumulative ligand affinities, K_B. The ligands were connected by linkers modeled as harmonic bonds with a force constant of $k = 1$, and the distance between potential wells on the surface matched the equilibrium lengths of these linkers. This chart presents three one-dimensional slices of the two-dimensional data for the same systems shown in Figure 3.

Diffusion in systems not displaying pattern matching

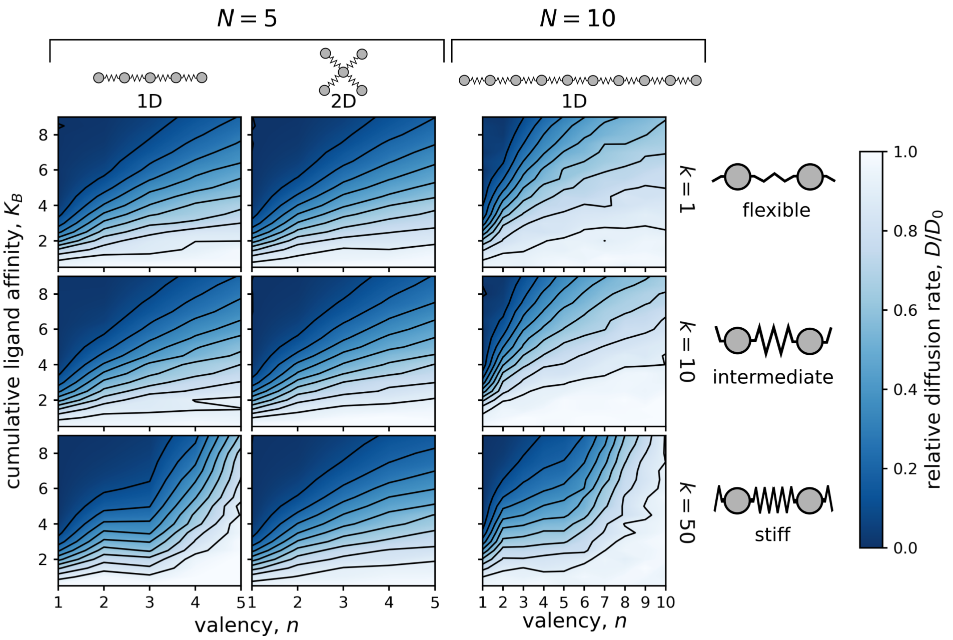

Figure S4: Dependence of the relative diffusion rate, D/D_0 on the valency, n, and the cumulative ligand affinity, K_B. Simulations were performed using particles consisting of either $N = 5$ (two columns on the left) or $N = 10$ ligands (column on the right) connected by flexible ($k = 1$; top row), intermediate ($k = 10$; middle row), or stiff ($k = 50$; bottom row) linkers. The distance between the surface potential wells, $\lambda/\lambda_{\text{eq}}$, was set to 0.7 (in contrast to $\lambda/\lambda_{\text{eq}}$ of 1.0 in Figure 3). Diffusion of particles with $N = 5$ was calculated in both 1D (linear chain) and 2D (star) geometries, while diffusion of particles with $N = 10$ was examined only in the 1D case (linear chain). Note that for stiffly linked ligands ($k = 50$), the 1D relative diffusion rate increases exponentially with increasing valency. This behavior is in contrast to what was observed in Figure 3 and it is the consequence of the wells distances not matching the length of the linkers ($\lambda/\lambda_{\text{eq}} = 0.7$). One simulation was run for each integer n and each increment of 0.5 in K_B.

Detailed analysis of pattern matching

As mentioned in the main text, we observed the lowest diffusion rate for particles whose linkers were slightly *shorter* than the distance between the surface potential wells. Specifically, the relative diffusion rate, represented by D/D_0, was found to be lower for systems with $\lambda/\lambda_{\text{eq}} = 1.1$ compared to systems with $\lambda/\lambda_{\text{eq}} = 1.0$. This trend was consistently observed across a wider range of particles with $N \in \{2 \ldots 5\}$ and $k = 10$ diffusing on a line (1D), as depicted in Figure S5 A.

For further analysis, we focused on the simplest case of a two-binding ligand particle, which was diffusing on a line (1D). In this case, the unnormalized (absolute) mean diffusion rates D were calculated as 0.176 ± 0.011 and 0.157 ± 0.011 arb.u. for $\lambda/\lambda_{\text{eq}} = 1.0$ and $\lambda/\lambda_{\text{eq}} = 1.1$, respectively. To gain insights into the underlying mechanisms, we calculated the potential energy surfaces for these two systems, considering the two degrees of freedom of the two-ligand particle, as depicted in Figure S5 B. Both potential energy surfaces exhibited characteristic diagonally repeating local minima. Surprisingly, as shown in S5 C, the potential energy barriers between these minima were lower for the system with slower diffusion ($\lambda/\lambda_{\text{eq}} = 1.1$), suggesting that the observed differences in diffusion rates were originating from entropic effects. Our hypothesis was that the faster diffusion for $\lambda/\lambda_{\text{eq}} = 1.0$ was due to the presence of four distinct paths that the ligand could take to transition from one local energy minimum to a neighboring minimum, as indicated by the white arrows in Figure S5 B. In contrast, the potential energy surface for $\lambda/\lambda_{\text{eq}} = 1.1$ only presented two energetically favorable paths that allowed the ligand to leave each minimum.

To test this hypothesis, we conducted additional simulations for each of the described systems (with $\lambda/\lambda_{\text{eq}}$ values of 1.0 and 1.1). In these simulations, we implemented a restriction to prevent the ligands from entering specific regions of the configuration space, as indicated by the white circles in S5 B. To achieve this, we applied a restraint in the following form:

$$u_{\text{res}} = \begin{cases} \infty & \text{if } \exists d \in D : d < 0.3 \\ 0 & \text{if } \forall d \in D : d \geq 0.3, \end{cases} \tag{S2}$$

where D is a set of distances between the ligands' coordinates x_i and x_j and the centers of the restricted areas, defined as

$$D = \left\{ \begin{matrix} \sqrt{[x_i - (n+0.2)\lambda]^2 + [x_j - (n+1.8)\lambda]^2}, \\ \sqrt{[x_i - (n+1.8)\lambda]^2 + [x_j - (n+0.2)\lambda]^2} \end{matrix} \mid n \in \mathbb{Z} \right\}. \tag{S3}$$

The applied restraint had a significant impact on the diffusion behavior of the particle with $\lambda/\lambda_{\text{eq}} = 1.0$. By obstructing half of the energetically most favorable paths, the diffusion rate of the particle was reduced by approximately 40% to 0.104 ± 0.008 arb.u. In contrast, the effect of the restraint on the system with $\lambda/\lambda_{\text{eq}} = 1.1$ was milder. It led to a modest decrease in the diffusion rate of about 16% to 0.132 ± 0.007 arb. u., as the particle already exhibited a preference for the unblocked paths.

This showed that if the same number of paths was available for the particle to take, the diffusion rates were consistent with the height of the potential energy barriers (i.e. lower potential energy barriers between the minima led to higher diffusion rate) and that the originally observed discrepancy was indeed introduced by entropic effects.

In the next two paragraphs, we attempt to provide an intuitive explanation for the existence of 4 or 2 energetically favorable paths. When the distance between the wells perfectly matched the length of the linker ($\lambda/\lambda_{\text{eq}} = 1.0$) and both ligands of the particle resided in neighboring potential wells, the linker would be in equilibrium, meaning there would be no tension in the linker. In such

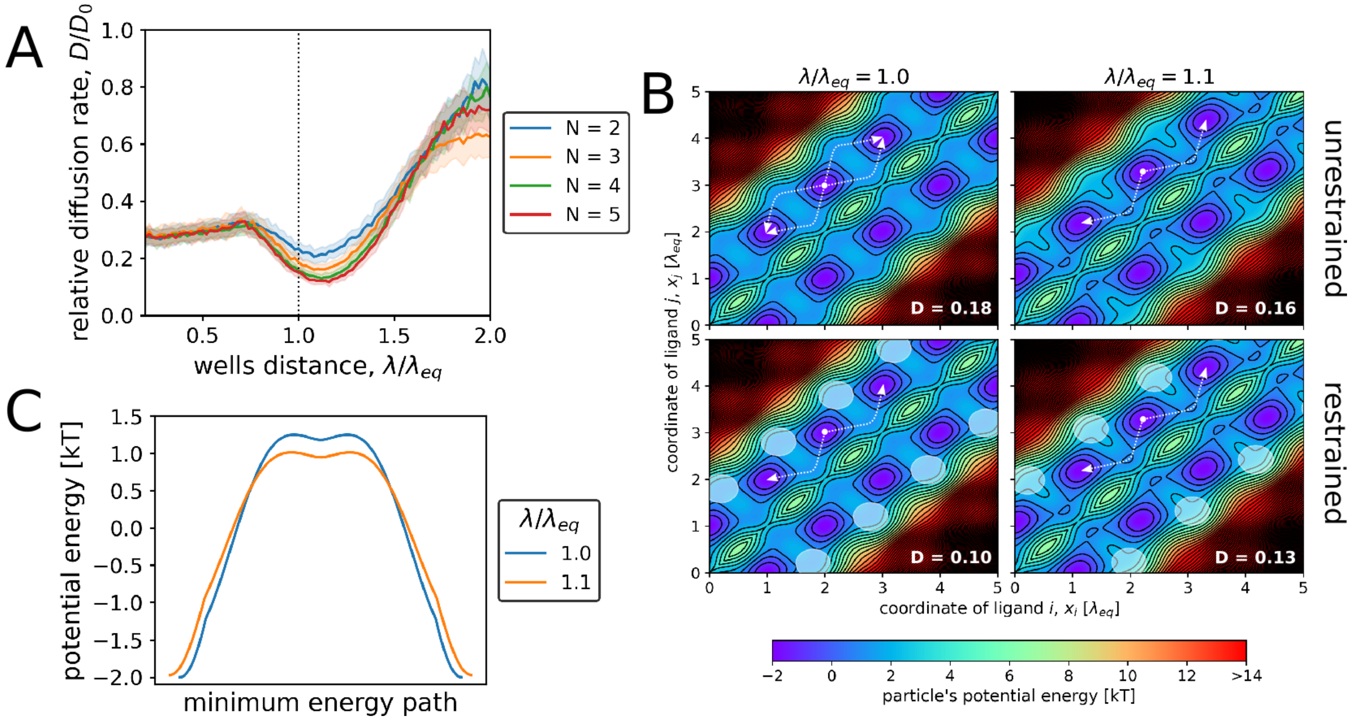

Figure S5: A. Dependence of 1D relative diffusion rate, D/D_0, on the distance between the surface potential wells, $\lambda/\lambda_{\mathrm{eq}}$, for particles composed of varying number of ligands, N. All ligands were binding ($n = N$) and all ligands were connected into a linear chain using linkers with a force constant, k, of 10. One simulation was run for each each increment of 0.02 in $\lambda/\lambda_{\mathrm{eq}}$. B. Slices of potential energy surfaces for a particle composed of two intermediately linked (k = 10) binding ligands in a system with $\lambda/\lambda_{\mathrm{eq}} = 1.0$ (left column) or $\lambda/\lambda_{\mathrm{eq}} = 1.1$ (right column). Potential energy is shown as a function of the coordinates of the particle's ligands. The white arrows show energetically favorable paths the particle can take to move between neighbouring potential energy minima. In the bottom row, the white circles show areas that the particle was restricted from in the restrained simulations. In the lower right corner of each chart, we show the unnormalized diffusion rate calculated for this potential energy surface (in arb. u.). C. Potential energy profiles of the particle along the identified paths between two neighbouring energy minima, calculated for $\lambda/\lambda_{\mathrm{eq}} = 1.0$ and for $\lambda/\lambda_{\mathrm{eq}} = 1.1$. The energies were obtained from the analytically calculated potential energy surfaces using the MEPSAnd script version 1.6 (available from bioweb.cbm.uam.es/software/MEPSAnd).

case, the particle's diffusion could be initiated by either ligand moving in either direction, resulting in the stretching or compressing of the linker. Thus, there were four possible ways for the particle to leave the energy minimum, as depicted in the potential energy surface for $\lambda/\lambda_{\mathrm{eq}} = 1.0$.

In contrast, if the linker was slightly shorter than the distance between the wells ($\lambda/\lambda_{\mathrm{eq}} = 1.1$) and both ligands of the particle were located in neighboring potential wells, the linker was already slightly stretched, creating tension that forced the ligands closer together. Consequently, in such scenario, the particle's diffusion could not be initiated by either ligand moving *away* from the other, as this would involve energetically unfavorable stretching of the already stretched linker. Instead, the process of the particle leaving the energy minimum had to be triggered by either of the ligands moving *closer* to the other, bringing the linker closer to equilibrium. Therefore, the number of available paths is reduced to two, as illustrated in the potential energy surface for $\lambda/\lambda_{\mathrm{eq}} = 1.1$.

A similar mechanism is likely to apply also to particles composed of a larger number of ligands, which could explain the unexpected slowdown in diffusion at $\lambda/\lambda_{\mathrm{eq}} = 1.1$.

Binding free energy

We calculated the binding free energy for various one-dimensional systems. During the simulation run, we collected the positions of each ligand along a virtual z-axis. This position was calculated as

$$z_i = \sin\left[2\pi\left(\frac{x_i}{\lambda} - \frac{1}{4}\right)\right], \tag{S4}$$

where z_i represents the z-axis position of ligand i, and the other variables and constants are defined in Equation 1. A z-axis position was calculated and stored every 100 Monte Carlo sweeps (i.e., $100N$ Monte Carlo steps, where N is the number of ligands in the particle) during the production phase of the simulation. The simulation lengths were the same as described in the Methods section of the main text.

A histogram with two bins, corresponding to the "bound" ($z < 0$) and "free" ($z \geq 0$) states, was constructed from the collected z-axis positions.

The classification of the entire particle as "bound" or "free" was based on the state of its ligands. If all ligands were classified as "free", the particle was also classified as "free". In all other cases, the particle was classified as "bound". The free energy for each state was then calculated as $F = -\log W$, where W is the number of samples in the corresponding bin. The binding free energy, ΔF_B, was calculated as $F_{\text{bound}} - F_{\text{free}}$. See Figure S6 A for graphical depiction of the states.

We calculated the binding free energy of various 5-ligand particles, for which the 1D diffusion rates are presented in Figure 3. As shown in Figure S6 B, we found that the redistribution of the cumulative ligand affinity of the particle can affect the binding free energy. For particles with flexible and intermediate linkers ($k = 1$ and $k = 10$), we observed that the binding free energy becomes slightly less negative (indicating weaker binding) with increasing valency, n, of the particle. In contrast, the binding free energy remains constant for particles with stiff linkers ($k = 50$).

To verify that the conclusions presented in the main text are not merely artifacts of the non-constant binding free energy, we conducted simulations in which the binding affinities of individual ligands were carefully adjusted to maintain a constant binding free energy (see Table S1 for more information about the adjustment). As shown in Figure S7, keeping the binding free energy constant produces trends in diffusion rates that are consistent with those described in the main text (compare with the 1D systems with $k = 1$ and $k = 10$ in Figure 3), although the rate of increase is slightly lower, as expected. Consistent with previous results, the diffusion rate increases rapidly with increasing valency for particles with flexible linkers ($k = 1$) and more slowly for particles with intermediate linkers ($k = 10$), due to the pattern matching effect described in the main text.

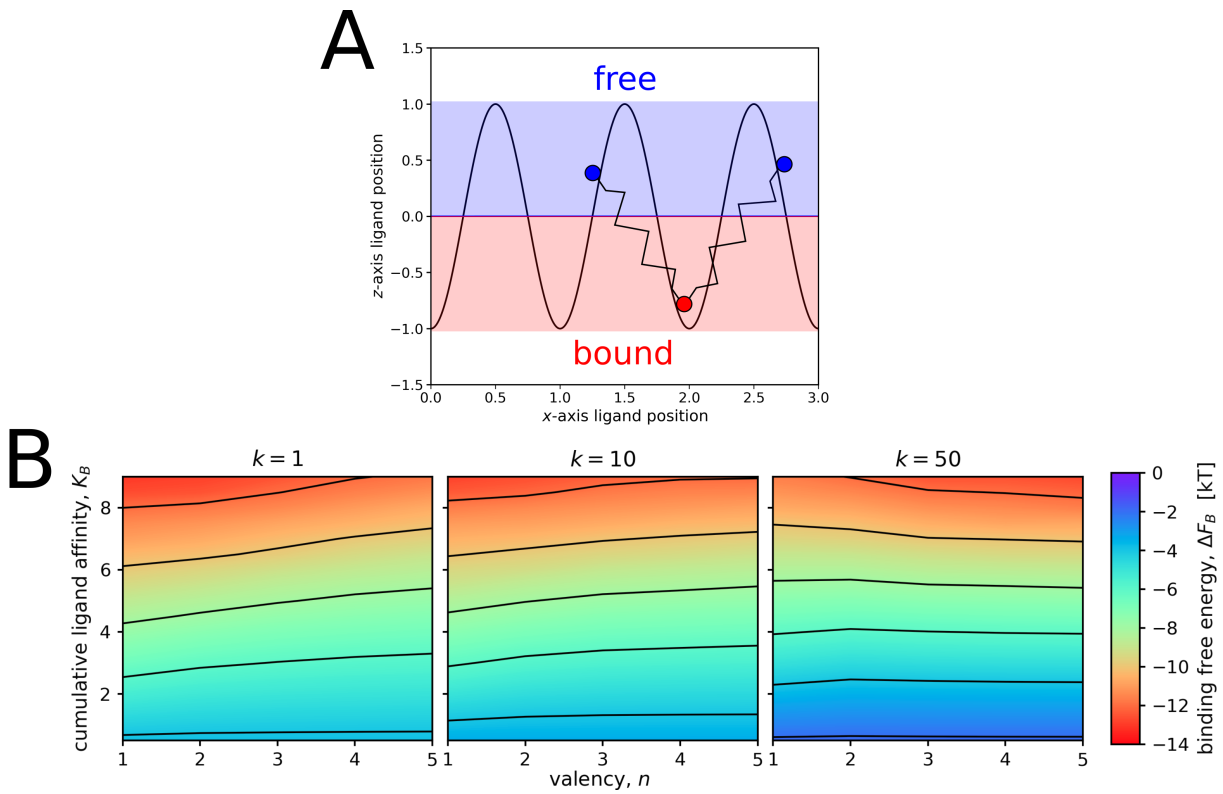

Figure S6: A. Sine wave defining both the surface potential and the virtual surface of the one-dimensional systems in our model, with illustrations of the "free" and "bound" states of a ligand. The figure also shows a three-ligand particle with two "free" ligands and one "bound" ligand. A ligand is considered "free" if its z-axis position is 0.0 or higher; otherwise, it is considered "bound". The particle is classified as "free" only if all its ligands are "free". B. Dependence of the binding free energy, ΔF_B, of the particles on the valency, n, and the cumulative ligand affinity, K_B. Simulations were performed on particles consisting of $N = 5$ ligands connected by flexible ($k = 1$; left), intermediate ($k = 10$; middle), or stiff ($k = 50$; right) linkers. The particles diffused in one dimension, with the distance between the surface potential wells matching the equilibrium linker lengths ($\lambda/\lambda_{\mathrm{eq}}$). One simulation was run for each integer value of n and each increment of 0.5 in K_B. The code used to perform the simulations with free energy calculations is available from doi.org/10.5281/zenodo.13771494.

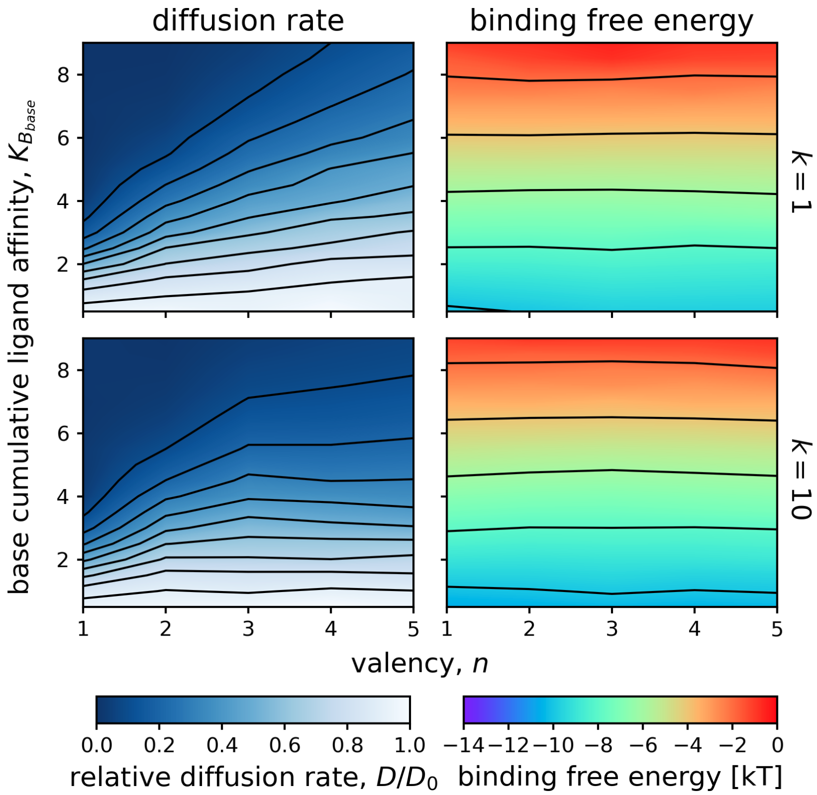

Figure S7: Left column. The dependence of the relative diffusion rate on the valency, n, and the "base" cumulative ligand affinity, $K_{B_{\mathrm{base}}}$, of the particle. The actual cumulative ligand affinity, K_B, varied slightly for different valencies to ensure a constant binding free energy, ΔF_B (see Table S1 for information about the actual ligand affinities used). Data for particles with flexible linkers ($k = 1$) are presented in the top row, while data for particles with intermediate linkers ($k = 10$) are shown in the bottom row. Compare these results with the 1D, $k = 1$ and $k = 10$ charts in Figure 3. Right column. Binding free energy calculated for the corresponding systems. The binding free energy remained approximately constant even as valency increased, achieved by redistributing additional binding affinity among the binding ligands of the particle as valency increased (see Table S1).

Table S1: Cumulative ligand affinities, K_B, distributed between the ligands of various particles to achieve a constant binding free energy (see results in Figure S7).

k	$K_{B_{\text{base}}}$	n	K_B	k	$K_{B_{\text{base}}}$	n	K_B
1.0	0.5	1	0.5	1.0	5.0	1	5.0
		2	0.8			2	5.3
		3	1.1			3	5.6
		4	1.1			4	5.9
		5	1.1			5	6.2
1.0	1.0	1	1.0	1.0	5.5	1	5.5
		2	1.3			2	5.8
		3	1.6			3	6.1
		4	1.6			4	6.4
		5	1.6			5	6.7
1.0	1.5	1	1.5	1.0	6.0	1	6.0
		2	1.8			2	6.3
		3	2.1			3	6.6
		4	2.1			4	6.9
		5	2.1			5	7.2
1.0	2.0	1	2.0	1.0	6.5	1	6.5
		2	2.3			2	6.8
		3	2.6			3	7.1
		4	2.6			4	7.4
		5	2.6			5	7.7
1.0	2.5	1	2.5	1.0	7.0	1	7.0
		2	2.8			2	7.3
		3	3.1			3	7.6
		4	3.1			4	7.9
		5	3.3			5	8.2
1.0	3.0	1	3.0	1.0	7.5	1	7.5
		2	3.3			2	7.8
		3	3.6			3	8.1
		4	3.6			4	8.4
		5	3.8			5	8.7
1.0	3.5	1	3.5	1.0	8.0	1	8.0
		2	3.8			2	8.3
		3	4.1			3	8.6
		4	4.4			4	8.9
		5	4.7			5	9.2
1.0	4.0	1	4.0	1.0	8.5	1	8.5
		2	4.3			2	8.8
		3	4.6			3	9.1
		4	4.9			4	9.4
		5	5.2			5	9.7
1.0	4.5	1	4.5	1.0	9.0	1	9.0
		2	4.8			2	9.3
		3	5.1			3	9.6
		4	5.4			4	9.9
		5	5.7			5	10.2

Table continues

k	$K_{B_{\text{base}}}$	n	K_B	k	$K_{B_{\text{base}}}$	n	K_B
10.0	0.5	1	0.5	10.0	5.0	1	5.0
		2	0.7			2	5.2
		3	0.9			3	5.4
		4	0.8			4	5.6
		5	0.9			5	5.8
10.0	1.0	1	1.0	10.0	5.5	1	5.5
		2	1.2			2	5.7
		3	1.4			3	5.9
		4	1.3			4	6.1
		5	1.4			5	6.3
10.0	1.5	1	1.5	10.0	6.0	1	6.0
		2	1.7			2	6.2
		3	1.9			3	6.4
		4	1.8			4	6.6
		5	1.9			5	6.8
10.0	2.0	1	2.0	10.0	6.5	1	6.5
		2	2.2			2	6.7
		3	2.4			3	6.9
		4	2.5			4	7.1
		5	2.6			5	7.3
10.0	2.5	1	2.5	10.0	7.0	1	7.0
		2	2.7			2	7.2
		3	2.9			3	7.4
		4	3.0			4	7.6
		5	3.1			5	7.8
10.0	3.0	1	3.0	10.0	7.5	1	7.5
		2	3.2			2	7.7
		3	3.4			3	7.9
		4	3.5			4	8.1
		5	3.6			5	8.3
10.0	3.5	1	3.5	10.0	8.0	1	8.0
		2	3.7			2	8.2
		3	3.9			3	8.4
		4	4.1			4	8.6
		5	4.3			5	8.8
10.0	4.0	1	4.0	10.0	8.5	1	8.5
		2	4.2			2	8.7
		3	4.4			3	8.9
		4	4.6			4	9.1
		5	4.8			5	9.3
10.0	4.5	1	4.5	10.0	9.0	1	9.0
		2	4.7			2	9.2
		3	4.9			3	9.4
		4	5.1			4	9.6
		5	5.3			5	9.8

Effect of ligand distribution on diffusion

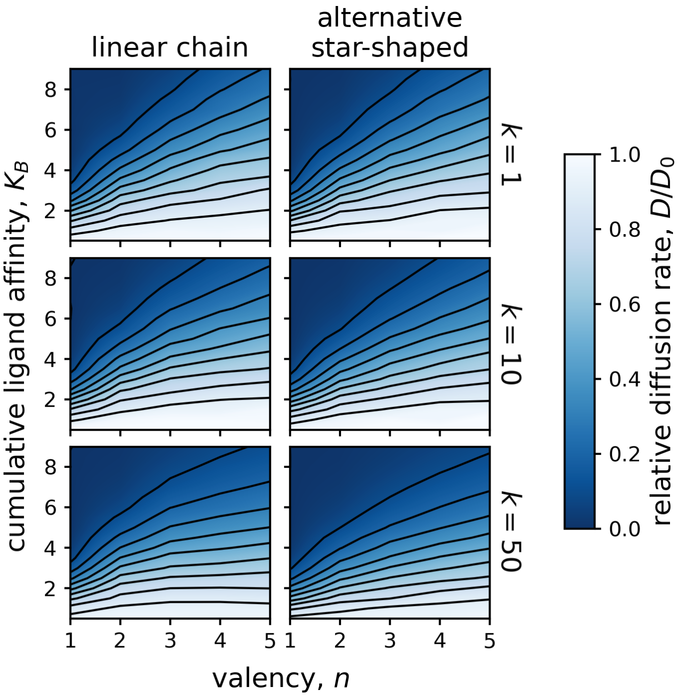

Figure S8: Dependence of the relative diffusion rate, D/D_0, on the valency, n, and the cumulative ligand affinity, K_B, for $N = 5$ particles with different geometries diffusing on a plane (2D). The left column shows results for particles with ligands arranged in a linear chain, while the right column shows results for alternative star-shaped particles, where the central ligand always interacted with the surface potential (unless $n = 0$). Note that for the standard star-shaped particles, the central ligand was always non-binding, unless $n = N$. See Table S2 for a detailed depiction of the geometries used in these simulations. The simulated particles consisted of flexibly ($k = 1$; top row), intermediately ($k = 10$; middle row), or stiffly linked ligands ($k = 50$; bottom row), and the distance between the surface potential wells matched the equilibrium linker lengths. Note that the overall trends in the diffusion rates matched the trends described in the main text for the standard distributions of the binding ligands. One simulation was run for each integer n and each increment of 0.5 in K_B.

Table S2: Distributions of ligands used for 2D simulations presented in Figure S8. ● corresponds to a ligand that interacts with the surface potential (binds to receptors), while ○ corresponds to a non-binding ligand.

	linear chain	alternative star-shaped
$n = 0$	○○○○○	○<br>○○○<br>○
$n = 1$	●○○○○	○<br>○●○<br>○
$n = 2$	●○○○●	○<br>●●○<br>○
$n = 3$	●○●○●	○<br>●●●<br>○
$n = 4$	●●●○●	●<br>●●●<br>○
$n = 5$	●●●●●	●<br>●●●<br>●

Diffusion with a chain move

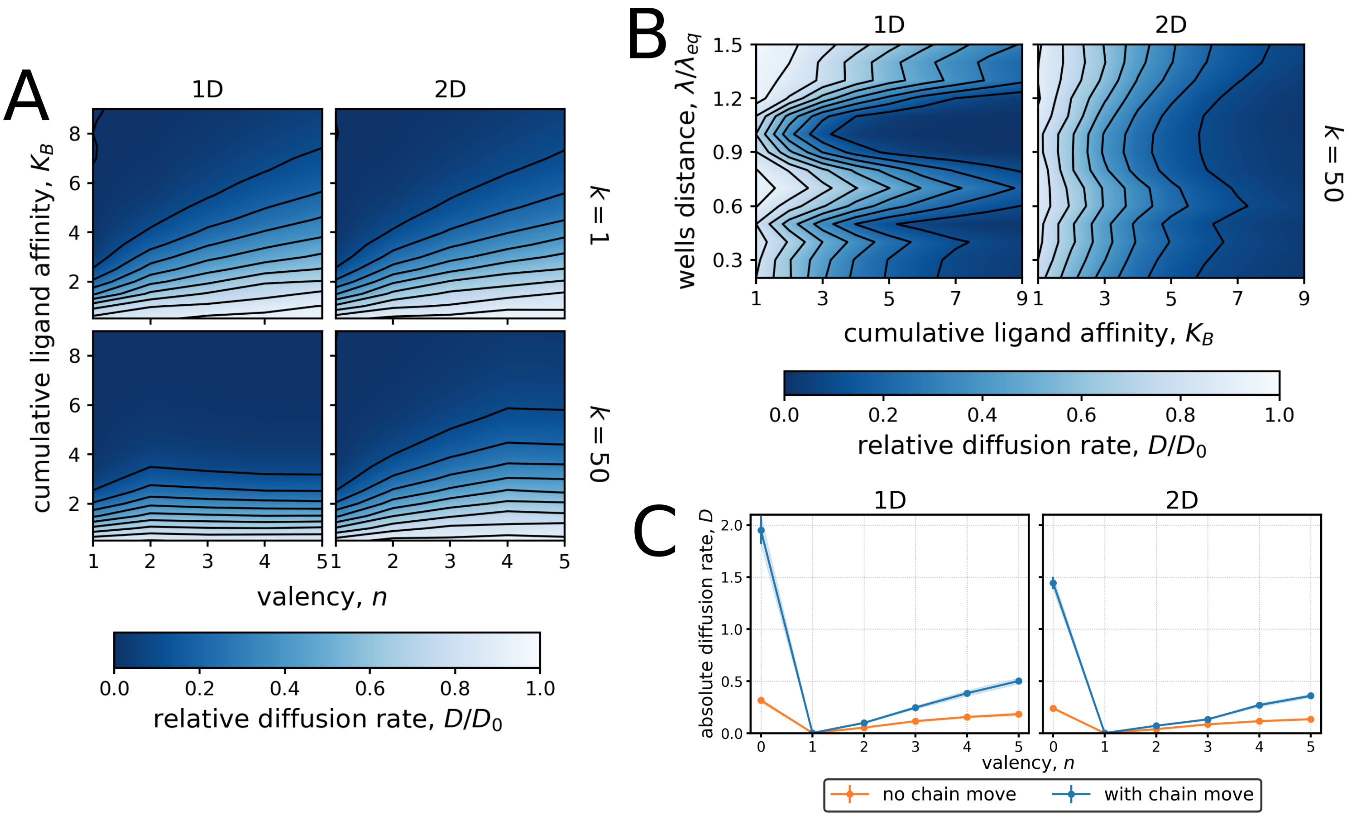

Figure S9: Diffusion with a chain move. The chain move involved moving all connected ligands in the same direction by the same distance. The maximal displacement was $0.1\lambda_{eq}$, the same as for the standard translation move. A chain move was attempted on average once every sweep (once every N Monte Carlo steps), replacing a standard translation move of a randomly selected ligand. For particles with 5 ligands ($N = 5$), which are presented here, this resulted in 20% of the attempted moves being chain moves. The code used to perform the simulations with the chain move is available from doi.org/10.5281/zenodo.10877722. A. Dependence of the relative diffusion rate, D/D_0, on the valency, n, and the cumulative ligand affinity. The ligands were connected either with flexible ($k = 1$; top row) or stiff linkers ($k = 50$; bottom row). The distance between the surface potential wells matched the equilibrium linker lengths. Diffusion was calculated for both 1D (linear chain; left column) and 2D (star-shaped; right column) geometries. One simulation was run for each integer n and each increment of 0.5 in K_B. Compare with relative diffusion rates in the top and bottom rows of Figure 3 for the $N = 5$ case. B. Dependence of the relative diffusion rate, D/D_0, on the cumulative ligand affinity, K_B, and the distance between the potential wells, λ/λ_{eq}. The ligands, all of which were binding, were arranged either as a linear chain (1D; left) or as a star (2D; right) and were connected with stiff linkers ($k = 50$). One simulation was run for each increment of 0.5 in K_B and each increment of 0.1 in λ/λ_{eq}. Compare with relative diffusion rates in the bottom row of Figure 4. C. Comparison of absolute diffusion rates calculated for several systems without the chain move (red) and with the chain move (blue). The linkers were flexible ($k = 1$) and the cumulative ligand affinity, K_B, was 5.0. Diffusion was calculated for both 1D (left) and 2D (right) geometries. Other parameters are described in panel A.

Diffusion on step surface potential

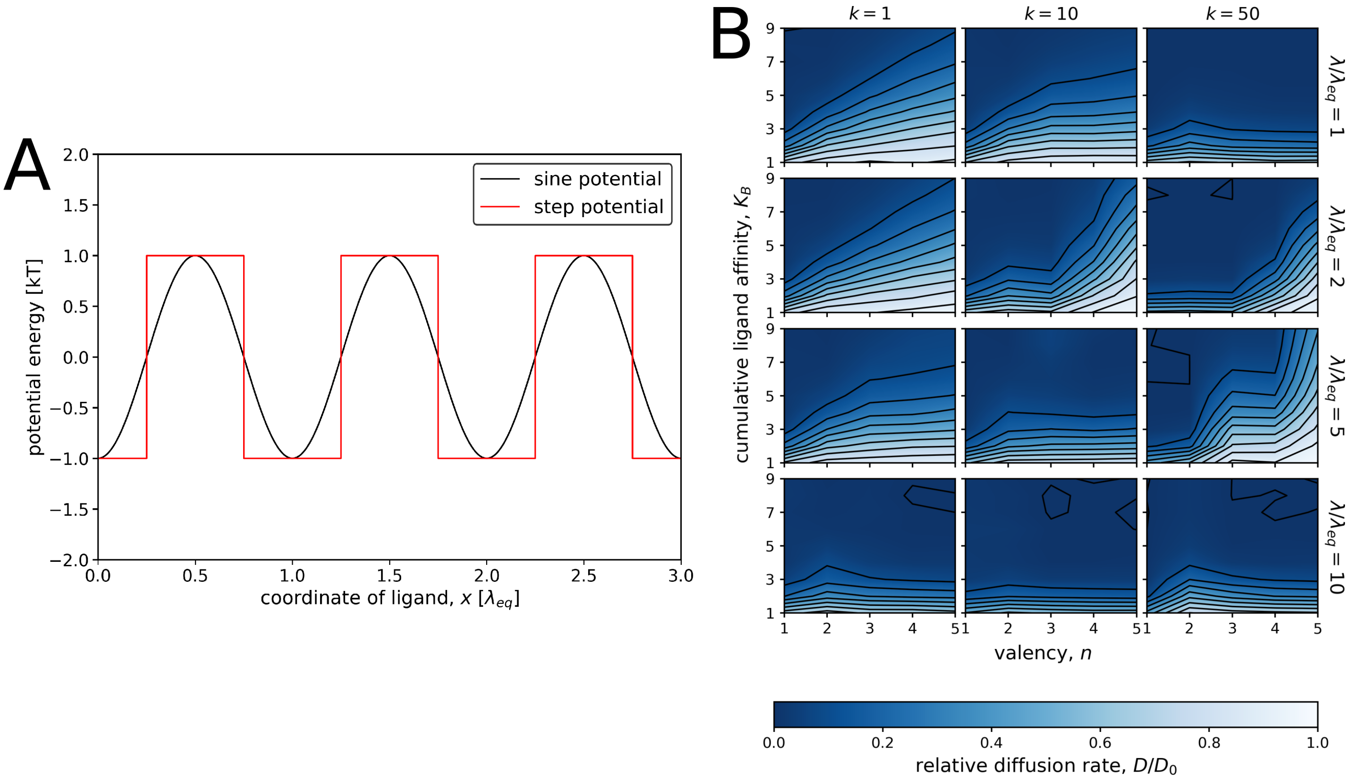

Figure S10: A. 1D sine surface potential used in the main text as given by the equation in Figure 1 (in black) compared to 1D step surface potential used in the panel B and given by Equation (S5) (in red). B. Dependence of the 1D relative diffusion rate, D/D_0, on the valency, n, and the cumulative ligand affinity, K_B, for $N = 5$ particles connected by flexible ($k = 1$; left column), intermediate ($k = 10$; middle column), or stiff ($k = 50$; right column) bonds. The particles diffused on a step surface potential with wells distances of 1 (top row), 2 (second row), 5 (third row), or 10 (last row). One simulation was run for each integer n and K_B. Increasing wells distance expands step widths. For $\lambda/\lambda_{\mathrm{eq}} = 1$, particle behavior mirrored that on a sine surface potential with equivalent wells distance (see Figure 3). Generally, trends remained consistent with the sine surface potential, with greater valency increasing diffusion. Some deviations occurred due to pattern matching. At $\lambda/\lambda_{\mathrm{eq}} = 10$, valency stopped influencing diffusion across all bond stiffnesses, as wide energy barriers required all particle ligands to cross each barrier simultaneously, making the diffusion dependent almost entirely on the cumulative ligand affinity of the particle. The code used to perform the simulations with step surface potential is available from doi.org/10.5281/zenodo.10054283.

$$u_i = A\ \mathrm{sgn}\left\{\sin\left[2\pi\left(\frac{x_i}{\lambda} - \frac{1}{4}\right)\right]\right\} \tag{S5}$$
